# Next-Generation Sequencing Techniques Reveal that Genomic Imprinting Is Absent in Day-Old *Gallus gallus domesticus* Brains

**DOI:** 10.1371/journal.pone.0132345

**Published:** 2015-07-10

**Authors:** Qiong Wang, Kaiyang Li, Daixi Zhang, Junying Li, Guiyun Xu, Jiangxia Zheng, Ning Yang, Lujiang Qu

**Affiliations:** Department of Animal Genetics and Breeding, National Engineering Laboratory for Animal Breeding, College of Animal Science and Technology, China Agricultural University, Beijing, China; University of Sydney, AUSTRALIA

## Abstract

Genomic imprinting is a phenomenon characterized by parent-of-origin-specific gene expression. While widely documented in viviparous mammals and plants, imprinting in oviparous birds remains controversial. Because genomic imprinting is temporal- and tissue-specific, we investigated this phenomenon only in the brain tissues of 1-day-old chickens (*Gallus gallus*). We used next-generation sequencing technology to compare four transcriptomes pooled from 11 chickens, generated from reciprocally crossed families, to the DNA sequences of their parents. Candidate imprinted genes were then selected from these sequence alignments and subjected to verification experiments that excluded all but one SNP. Subsequent experiments performed with two new sets of reciprocally crossed families resulted in the exclusion of that candidate SNP as well. Attempts to find evidence of genomic imprinting from long non-coding RNAs yielded negative results. We therefore conclude that genomic imprinting is absent in the brains of 1-day-old chickens. However, due to the temporal and tissue specificity of imprinting, our results cannot be extended to all growth stages and tissue types.

## Introduction

Genomic imprinting is an epigenetic phenomenon in which certain genes are expressed in a parent-of-origin-specific manner [1–3]. In other words, genes exhibit monoallelic or preferential allelic expression: maternal imprinting silences alleles from the mother, resulting in the predominant expression of paternal alleles, while paternal imprinting is the reverse, where maternal alleles are expressed predominantly.

The parental conflict hypothesis is one of the most plausible explanations for genomic imprinting, offering an evolutionary advantage for this epigenetic mechanism. Sexual conflict between the parents results in the biased expression of genes that affect maternal resource allocation. Specifically, maternal genes are selected to conserve sufficient resources for both the mother and her progeny, while paternal genes are selected to use resources in a way that maximizes offspring growth at the expense of the mother [4,5]. Under the logic of this hypothesis, there is little selective pressure for genomic imprinting in oviparous animals such as birds, because their embryos grow and develop in eggs. Thus, resource allocation to offspring is more equalized between mothers and fathers, resulting in less sexual conflict.

Genomic imprinting is very well documented in mammals and plants [6–14], although equivocal in insect models such as *Drosophila* [15,16]. Notably, some indirect evidence for imprinting has been found in birds, specifically chickens, suggesting that the parental conflict hypothesis may not fully explain this phenomenon. Previous studies on chickens have found parent-of-origin-specific quantitative trait loci (QTL) that either correspond to orthologous imprinted regions in human and mouse genomes, or are connected with economically important, typically imprinted traits in mammals [17]. As yet, however, researchers have been unable to determine whether these parent-specific QTL are actually dependent on imprinting, or whether other mechanisms are involved.

The recent advances in next-generation sequencing (NGS) technologies, including transcriptome sequencing (RNA-seq), now make answering such questions possible [18]. In fact, a recent study using NGS reported the lack of genomic imprinting in chicken embryos at 4.5 days [19]. However, because the analysis was performed using whole embryos, and genomic imprinting is both temporally and tissue-specific [1], such results do not fully exclude the possibility of imprinting in chickens. Conflicting parent allele-specific expression between different tissues, for example, may cancel each other out when measuring gene expression in the entire embryo.

Gene imprinting is a special case of allele-specific expression [20], and previously, we verified the tissue specificity of allele-specific expression in the chicken [21]. In this study, we chose to use brain tissue from 1-day-old chickens to investigate whether genomic imprinting is present in an oviparous animal. Brain tissue is ideal for our study because numerous genes are expressed in the brain [22], and it is commonly used for similar research. Additionally, imprinted genes have been implicated in neurodevelopment [23]. Coupled with our sample collection from the developmentally important 1-day-old stage, we believe our study will contribute to a further understanding of neurological ontogeny in the brain.

## Methods

### Ethics statement

Animal experiments were approved by the Animal Care and Use Committee of the China Agricultural University (Approval ID: XXCB-20090209). Animals were fed and handled according to the regulations and guidelines established by this committee, and all efforts were made to minimize suffering.

### Reciprocal design

To determine whether genomic imprinting exists in 1-day-old chicken brains, we compared the allelic-bias of four transcriptome libraries pooled from 11 chickens, descended from two reciprocally crossed families, to the DNA sequences of their parents.

**Fig 1:**
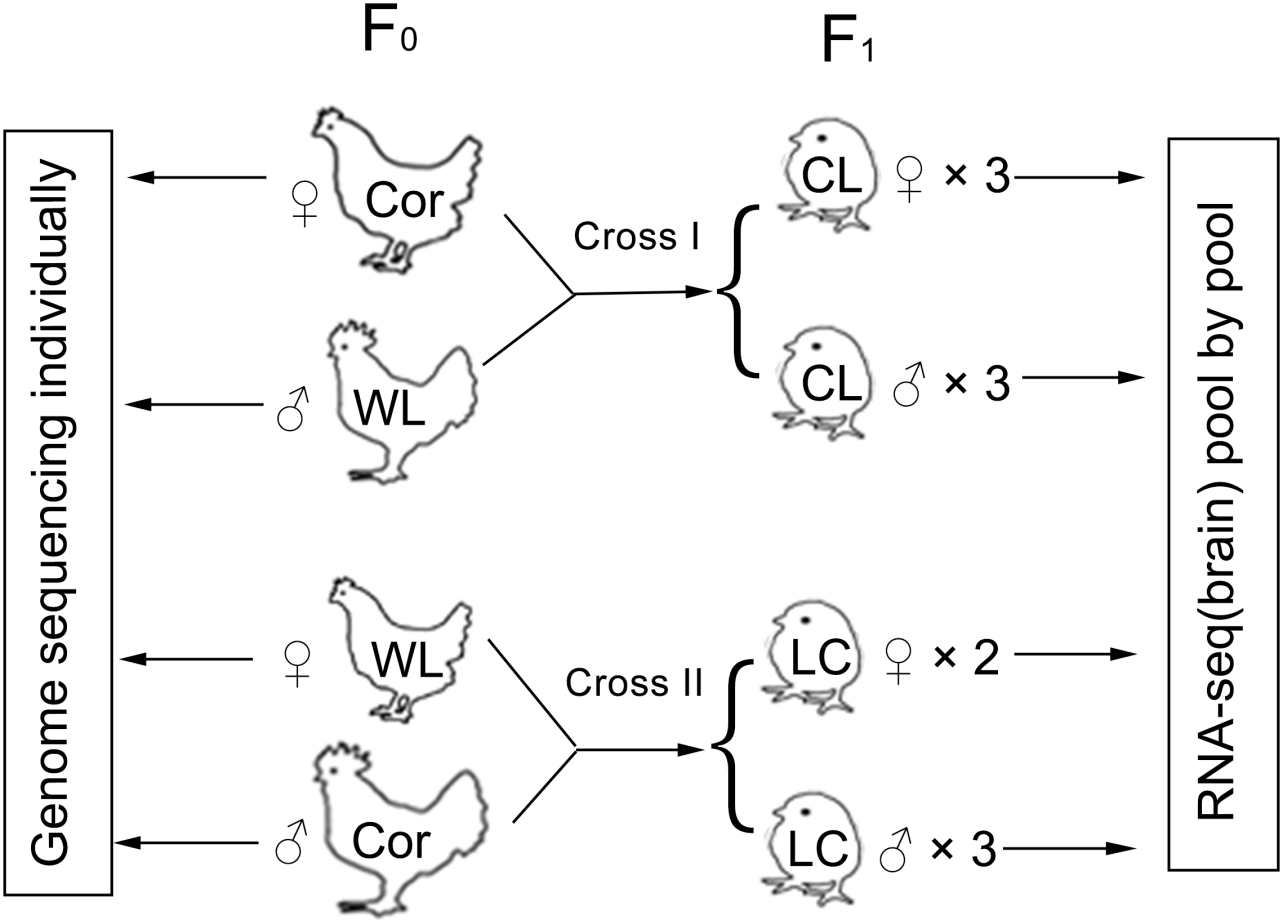
Reciprocal design. We reciprocally crossed two inbred strains, *Cornish* (Cor) and *White Leghorn* (WL), to generate progenies. For each cross, the brains of 1-day-old chickens from each sex were collected and total RNA was extracted from the tissue samples. Same-sex RNA samples from the same family were pooled to establish an RNA-seq library. The male and female pools within the same family were regarded as biological replicates. DNA samples of four parents from each family were used for whole-genome re-sequencing.

Two inbred strains (*Cornish*: Cor and *White Leghorn*: WL) were chosen as the reciprocally crossed parents (cross I: female Cor × male WL, cross II: female WL × male Cor) (Fig 1). Eleven 1-day-old chickens were euthanized with carbon dioxide and their brains collected (three females and three males in cross I, two females and three males in cross II because only two females were available). Total RNA was extracted from the brain tissue and all same-sex samples of the same family were pooled to establish an RNA-seq library. The pools of each family (that is, the female and the male pool) were used as two biological replicates of that family. DNA samples of four parents from each family were used for whole-genome re-sequencing.

### Data acquisition and analysis

Whole-genome sequencing of parent genomes and RNA-seq of offspring genomes were performed using an Illumina HiSeq 2500 sequencer. Library construction and sequencing were performed following manufacturer protocols (TruSeq DNA Sample Prep Kit, TruSeq RNA Sample Prep Kit, TruSeq PE Cluster Kit v3-cBot, and TruSeq SBS Kit v3). Paired-end reads of 100 bps were produced. The insert sizes of the re-sequencing and RNA-seq libraries were 300 bps and 330 bps, respectively.

Using our re-sequenced genome and the Galgal4 reference genome (from the Ensembl Genome Browser), we simulated Cor and WL genomes. We then used two programs, the Burrows-Wheeler Aligner’s Smith-Waterman Alignment tool [24] and the Genome Analysis Toolkit [25], to align the two simulated genomes and look for homozygous SNPs. Specifically, we verified that SNPs at a particular locus were the same within the two parent strains and different between them. We adopted two standards for filtering homozygous SNPs between the parent strains: a loose standard of higher than 2× sequencing depth and a general standard of higher than 10× depth. The loose standard was implemented first, to avoid missing any potential imprinted genes. Under both standards, reads should be the same at every homozygous SNP locus across the two sets of parental re-sequencing data.

Next, we identified SNPs exhibiting parent-of-origin allelic expression in the reciprocal crosses with TopHat software [26], which allows us to align RNA-seq reads to the simulated parent genomes. Each SNP locus was required to have at least 10 reads aligned to the parental alleles in the transcriptome pool, thus deviating from a 1:1 ratio. We used a chi-square test, with an adjusted (via Bonferroni correction) P-value set at 0.05, to look for a significant deviation. Additionally, the cut-off for number of SNPs was set at less than two per 10 bases. We considered an SNP to be potentially imprinted if the percentage of reads including those SNPs is more than 75% aligned to either the maternal or the paternal allele in both reciprocal crosses. Further, the allelic ratio of the SNP was expected to be inverted between the two crosses.

To annotate the candidate SNPs and predict their function, we used the SnpEFF program [27], which categorizes genomic variants based on their locations. For SNPs that are located in or near genes, the high alignment percentage roughly reflects the expression levels of those genes. Thus, SNPs that appear to be imprinted may also indicate the presence of potentially imprinted genes. We quality-checked our reads using the FastQC tools in the StatsDB package [28].

Finally, we identified any long non-coding RNAs that potentially exhibit imprinting. We assembled transcripts from our simulated genomes using the Cufflinks program [29]. We found new, previously unannotated transcripts by comparing the assembly results to known transcripts. We excluded any new transcripts that lacked at least one exon and was less than 200 bps in size. We screened for candidate non-coding RNAs using the Coding Potential Calculator [30]. The resultant transcripts were further filtered through the NCBI-nr database [31,32] to ensure accuracy. Candidate imprinted SNPs were then mapped to long non-coding RNAs.

### Verification experiments

NGS techniques may occasionally provide false positives [33]. Thus, to validate the sequences and expression patterns of candidate imprinted genes, and to verify SNP homozygosity across our parental samples, we performed the following verification experiments.

#### Restriction endonuclease analysis

We obtained cDNA from the RNA pools of offspring using reverse transcription polymerase chain reaction (RT-PCR). We then amplified any candidate genes of the offspring and parents that contained a potentially imprinted SNP. We generally used CAPS (cleaved amplified polymorphic sequences) assays for our analyses. However, in cases where the SNPs were not located at cleavage sites, we designed primers to introduce restriction enzyme sites and ran dCAPS (derived cleaved amplified polymorphic sequences) assays [34]. Amplified PCR products were separated with gel electrophoresis.

#### Direct Sanger sequencing

Next, we genotyped our amplified cDNA and DNA products using Sanger sequencing, to exclude the possibility of incomplete digestion.

#### Pyrosequencing

We aimed to detect imbalanced allelic expression in the offspring of our reciprocal crosses, through the use of pyrosequencing with Pyromark Q96 (Qiagen). One candidate SNP in particular warranted more detailed investigation, due to its unusual properties (apparently sex-specific imprinting; described in the Results and Discussion section, under the subsection of “Verification experiments excluded all potential imprinted genes”). For this SNP, we analyzed brain cDNA samples (obtained from PT-PCR), and extracted RNA from the liver, heart, and pectorals of each offspring separately. We also verified the SNP on two other reciprocally crossed families (cross III and cross IV), where the four parents’ genotypes are homozygous at that SNP locus. Similar to the previous four crosses, these parent genotypes were confirmed using Sanger sequencing.

#### Monoclonal sequencing

Since some candidate SNPs are located in regions that are difficult to amplify and sequence directly, we performed monoclonal sequencing of the two candidate genes in which those SNPs are located. We connected a pMD19-T Vector to the target segment containing the candidate SNP. The vector was then transformed into *Escherichia coli* for amplification and sequencing of the target DNA segments.

## Results and Discussion

### Identification of candidate imprinted genes

We obtained a total of 412,605,954 reads (82.5 Gb) in the re-sequenced genome and 237,006,676 reads (47.4 Gb) in RNA-seq. The four RNA-seq libraries contained 10.2 Gb, 11.3 Gb, 11.8 Gb, and 14.1 Gb of reads. All libraries passed our quality control checks, and the base quality was very high, even at the end of reads (Fig A and B in S1 File).

Using the re-sequencing and RNA-seq data, we were able to identify many allele-specific expressed SNPs. Of the 825,211,908 reads in the re-sequencing data, 812,227,808 (98.43%) reads could be mapped to the Galgal4 reference genome. The mean sequence depth was 19.5×. We identified 726,884 SNPs and 422,693 SNPs under the loose and general standards, respectively. Under the general standard, SNPs that occurred between the two strains covered 4,960 genes, relative to a total of 17,108 genes in the reference genome.

**Fig 2:**
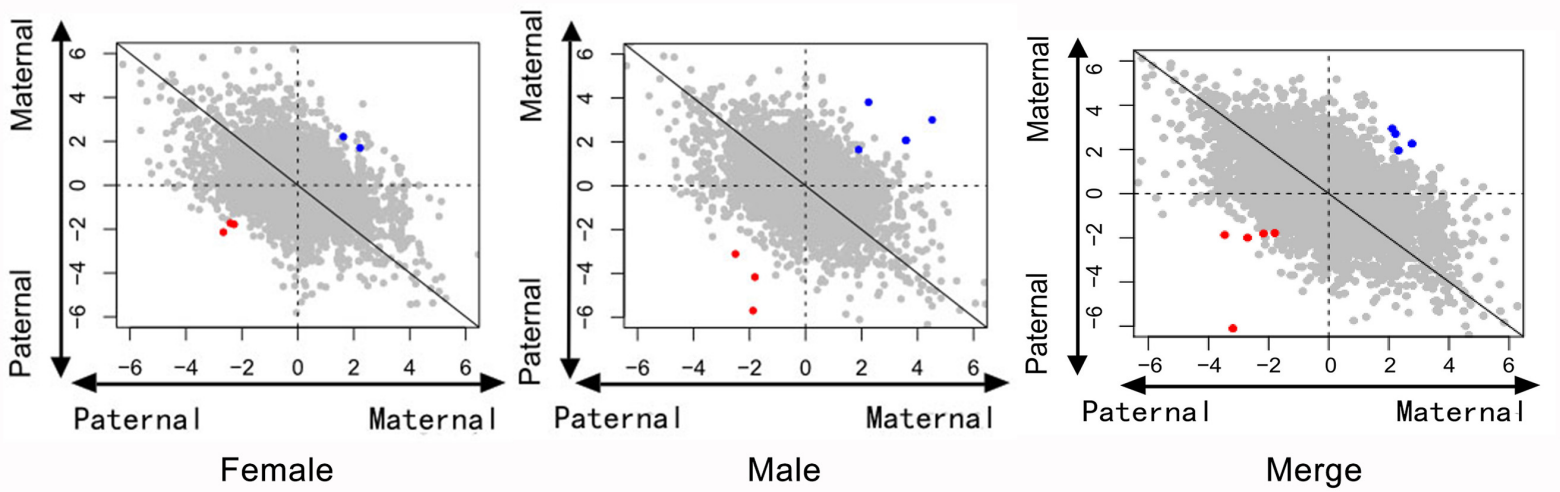
Ratio of reads aligned to parental genomes of all SNPs. Points in this figure reflect the ratio of reads aligned to the maternal versus the paternal genomes under the loose standard (SNP sequencing depth greater than 2×). The horizontal axis is log_2_ (m:f) in cross I, while the vertical axis is log_2_ (m:f) in cross II. Red points indicate the candidate maternal imprinted SNPs, while the blue points indicate candidate paternal imprinted SNPs.

Of these SNPs, we found several that potentially exhibited parent-of-origin effects. The proportion of reads aligned to the maternal genome was 0.5 at most SNP loci (chi-square test, p < 0.05. Fig C in S1 File). Under the general standard, we were only able to isolate one SNP in males, located on the autosomes. This SNP was also included under the looser standard, where we were able to select 1,478 SNPs in females (two from autosomes, 1,470 from the Z chromosome, and six unmapped to any chromosome) and 12 SNPs in males (eight from autosomes, one from the Z chromosome, and three unmapped to any chromosome) (Fig 2). Since the female is the heterogametic sex in birds, female Z chromosomes must come from the father, explaining why the 1,470 SNPs on the female Z chromosome exhibited paternal allelic expression. When we merged offspring female and male data, we found that 11 SNPs conformed to the loose standards (eight SNPs from autosomes and three SNPs unmapped to any chromosome).

The SNPs selected separately from the male and female data corresponded to eight potential imprinted genes on female autosomes and nine on male autosomes. Among the candidate genes in female autosomes, four exhibited maternal allelic expression, while the others exhibited paternal allelic expression. Among the male candidate genes, seven exhibited maternal expression, and the remaining two exhibited paternal expression. In the merged data, we found 10 potential imprinted genes, with five exhibiting maternal expression and the remainder exhibiting paternal expression. Among the maternally expressed genes in males, one was located on the Z chromosome. Information on these genes is summarized in Table 1.

**Table 1:** Candidate imprinted genes filtered by bioinformatics analysis of RNA-seq data.

No.	chromosome	SNP position	Gene name	Genotype	Effect	Imprinting type
1	12	19414668	VHL	T/G	Synonymous coding	Maternal
2	JH376330.1	16268	XLOC_047216	T/C	Non-synonymous coding	Paternal
3	16	59858	BFIV21	C/T	Synonymous coding	Paternal
4	JH375462.1	3673	XLOC_046149	A/T	Non-synonymous coding	Maternal
5	JH375607.1	9846	ENSGALG00000000194	C/T	Non-synonymous coding	Maternal
6	JH375607.1	13684	ENSGALG00000000194	T/C	Non-synonymous coding	Maternal
7	JH375628.1	3535	H3-IX	A/C	Non-synonymous coding	Maternal
8	JH375832.1	10462	XLOC_046722	A/G	Non-synonymous coding	Paternal
9	13	2890483	GABRP	A/T	Non-synonymous coding	Maternal
10	JH375328.1	424	XLOC_046087	A/G	Synonymous coding	Paternal
11	Z	943913	LMAN1	C/T	Stop codon gained	Paternal
12	2	4696581	ACAA1	T/C	Non-synonymous coding	Paternal
13	19	6454653	ZSWIM7	G/T	Non-synonymous coding	Paternal
14	JH375328.1	366	XLOC_046087	T/C	Non-synonymous coding	Paternal
15	5	23195463	PHF21A	G/A	Non-synonymous coding	Maternal
16*	8	6258175	FAM20B	A/G	Non-synonymous coding	Paternal

*No. 16 is the SNP filtered using the general standard.

In total, 2118 long, non-coding RNA transcripts were assembled from 1283 genes. Their average length was 2115 bps, and they contained 2.7 exons on average. However, none of these RNA transcripts showed any signs of imprinting.

### Verification experiments excluded all potential imprinted genes

Among the potential imprinted genes we identified, only two met the loose standards in both sexes (No. 1 *VHL* and No. 2 *XLOC_047216*). As imprinted genes may be expressed in a sex-specific pattern [35], we also verified 14 candidate genes that conformed to the loose standards in only one sex (S1 Table). The results of our verification experiments on these 16 genes are reported below.

**Fig 3:**
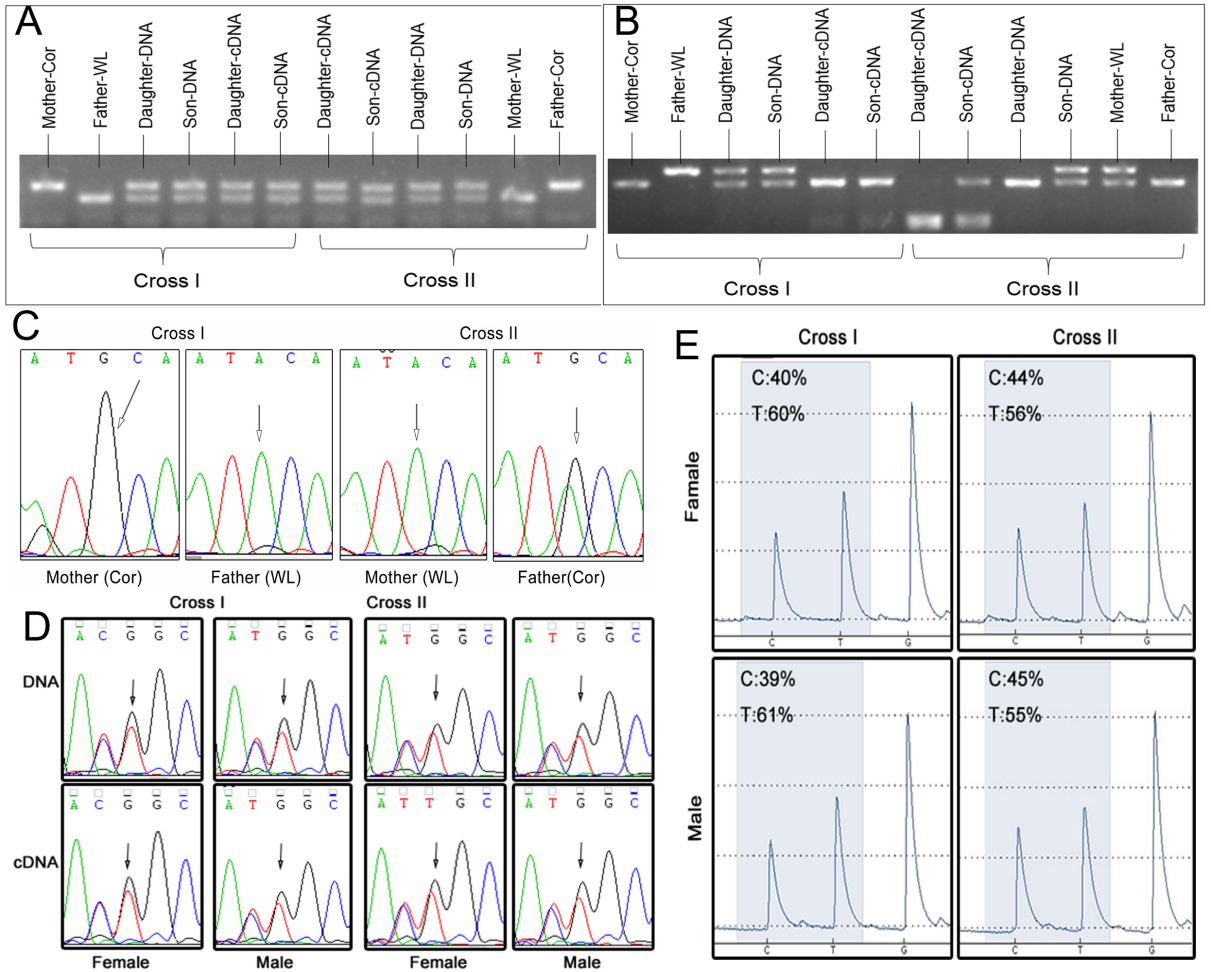
Verification experiments excluded most of the candidate imprinted SNPs. (A) Restriction endonuclease analysis. The cDNA pattern was similar to offspring DNA, which contained two bands of the same brightness (No. 4, gene *XLOC_046149*). (B) SNPs filtered using loose standards (sequencing depth > 2×) show heterozygosity in some parents (No. 5, *ENSGALG00000000194*). We designed different primers for our cDNA analysis versus our DNA analysis, due to the presence of an intron region near some SNPs in the DNA. (C) Direct Sanger sequencing of parental DNA confirming the heterozygosity of some SNPs. Sequencing results for gene *XLOC_046722* (No. 8) are shown; the SNP is the third base. (D) Direct Sanger sequencing of offspring DNA and cDNA. Expression of some genes does not show evidence of imprinting. Sequencing results for gene *ZSWIM7* (No. 13) are shown; the SNP is the third base. (E) Pyrosequencing results reveal that the gene expression patterns of most candidate genes do not show evidence of imprinting. Results for the cDNA of gene *ACAA1* (No. 12) are shown.

Our restriction endonuclease analysis revealed that four SNPs were heterozygous in certain parents (Fig 3A, 3B and Table 2). Three SNPs were confirmed heterozygous with Sanger sequencing of genes from the parent genomes (Fig 3C and Table 2), and two were confirmed with restriction endonuclease analysis. Pyrosequencing also verified five SNPs associated with four candidate genes, and monoclonal sequencing found that two SNPs associated with two candidate gene were heterozygous in the parent strains. In total, nine SNPs were confirmed heterozygous. Candidate genes associated with these SNPs were excluded.

**Table 2:** Results of verification experiments at each SNP locus.

No.	Sanger-offspring	Sanger-parents	Restriction enzyme digestion	Pyrosequencing	Result
1	\	Heterozygous*	\	\	Excluded
2	Inconformity of expression	Heterozygous*	\	\	Excluded
3	Inconformity of expression	Heterozygous	Parent heterozygous	Parent heterozygous	Excluded
4	Inconformity of expression	Coincident	Inconformity of expression	Inconformity of expression	Excluded
5	Inconformity of DNA	Heterozygous	Parent heterozygous	\	Excluded
**6**	**Coincident in female**	**Coincident**	**Coincident**	**Coincident in female**	**Suspicious (Sex-specific)**
7	Inconformity of expression	\	Parent heterozygous	\	Excluded
8	Inconformity of DNA	Heterozygous	\	Inconformity of expression, parent heterozygous	Excluded
9	Sequencing error	\	Inconformity of expression	\	Excluded
10	Inconformity of expression	\	Parent heterozygous	Inconformity of expression, parent heterozygous	Excluded
11	Inconformity of expression	Coincident	Inconformity of expression	Inconformity of expression	Excluded
12	Inconformity of expression	\	\	Inconformity of expression	Excluded
13	Inconformity of expression	Coincident	Inconformity of expression	\	Excluded
14	Inconformity of DNA	\	\	Inconformity of expression, parent heterozygous	Excluded
15	\	\	\	Parent heterozygous	Excluded
16	\	**Coincident**	\	Inconformity of expression	Excluded

The numbers in the first column correspond to those in Table 1.

*These two "Heterozygous" results were obtained via monoclonal sequencing.

"\"means that a verification experiment could not be performed at that SNP or results are not available.

"Inconformity of expression" indicates offspring expression patterns that were inconsistent with parent-of-origin specific expression. "Inconformity of DNA" indicates that the two kinds of base of SNPs in offspring DNA did not have equal content, which may be caused by heterozygosity in the parents. "Sequencing error" indicates the existence of complex structures beside the SNP that led to inaccurate Sanger sequencing results, which may have also caused errors in NGS.

We expected that offspring cDNA patterns in restriction endonuclease analyses may be similar to the pattern found in one of their parents, or appear as two bands that form a light and dark contrast. However, we found that all of our candidate genes exhibited preferential allelic expression instead of monoallelic expression. Thus, we were unable to compare the relative quantity of the original template using PCR amplification and gel electrophoresis. Offspring cDNA patterns for most candidate genes contained two bands of similar brightness. Direct Sanger sequencing results were similar to our findings using restriction endonuclease analysis. In sum, these analyses failed to find evidence of imprinting in candidate gene expression (Fig 3D and Table 2).

Monoclonal sequencing was performed on two candidate genes (No. 1 *VHL* and No. 2 *XLOC_047216*). The results revealed that one of them (No.1 *VHL*) was a heterozygote across the parent strains. The other candidate gene (No. 2 XLOC_047216) contained six bases that were either inserted or deleted next to the SNP. These indels may be the reason why the sequencing methods we chose were unsuitable, potentially causing errors in the reads statistic and the SNP filter.

**Fig 4:**
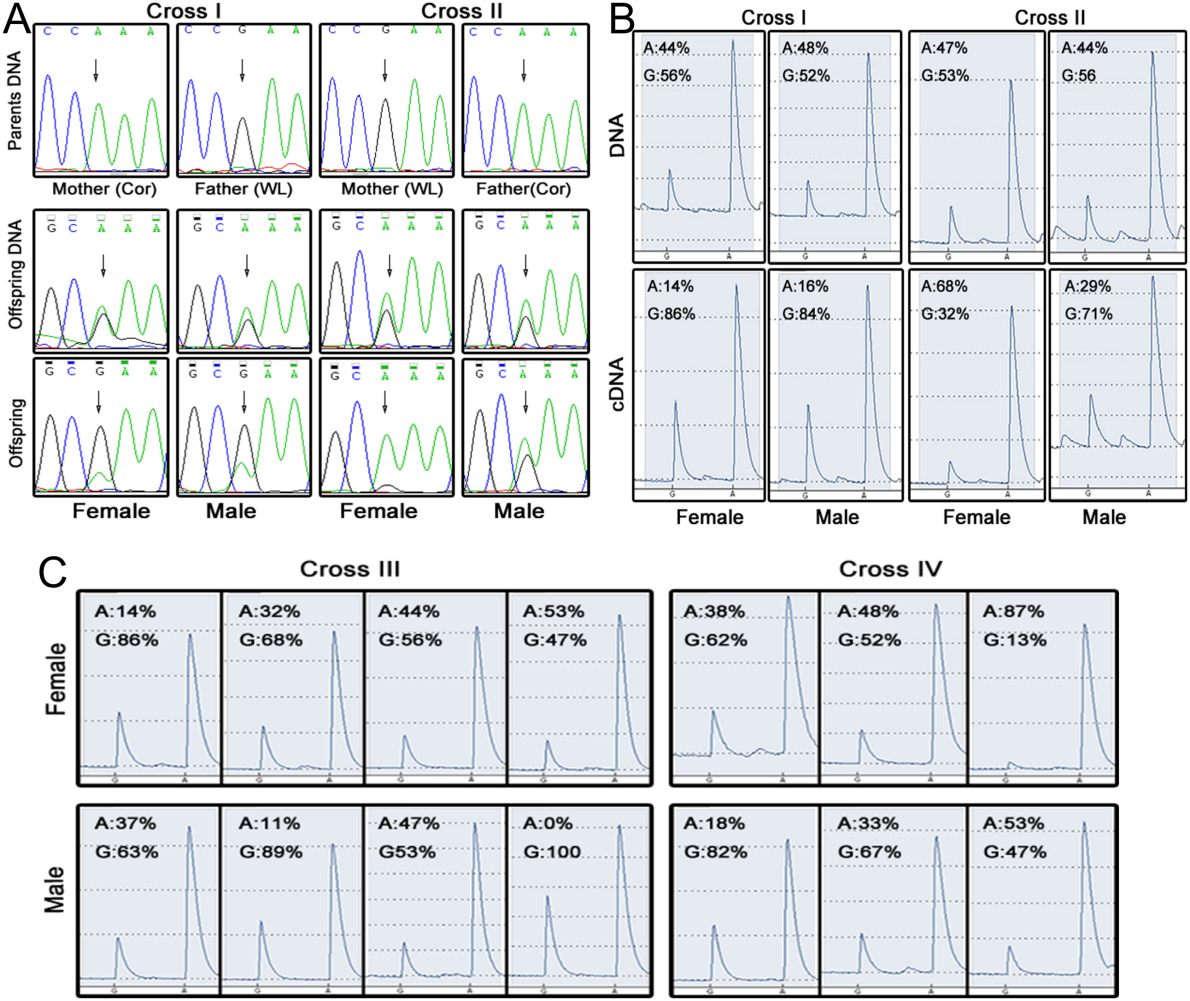
Results of further verification experiments on the SNP most likely to exhibit imprinting. The sequence near the SNP is CTCCCA/GAACGC. (A) This gene appeared to be expressed in a way indicative of genomic imprinting when examined with direct Sanger sequencing. However, the parent-of-origin characteristics were exhibited only in females. The relevant SNP is the third base. Differences between parent and offspring sequences in the first base are due to the introduction of mismatch bases in the offspring for dCAPS assays. (B) Pyrosequencing results are consistent with direct Sanger sequencing results. (C) Cross III and Cross IV, two different reciprocally crossed families, had parents homozygous at that SNP locus. This is in accordance with data from the previous four parents and was confirmed by Sanger sequencing. We detected the brain cDNA of offspring individually, using pyrosequencing. Neither males nor females in crosses III and IV exhibited parent-origin-specific gene expression patterns.

Finally, the results of our pyrosequencing revealed that the expression patterns of most candidate genes were not in accord with the characteristics of genomic imprinting (Fig 3E and Table 2). One gene (No. 6, gene *ENSGALG00000000194*) did appear to show expression patterns indicative of imprinting, but as it only exhibited parent-of-origin effects in females (Fig 4A and 4B), we believe it is more likely to be a sex-specific imprinted gene.

It is worth noting that there was another SNP (No. 5, *ENSGALG00000000194*) located in this sex-specific gene, although it was excluded due to heterozygosity in the parents. Nonetheless, the results of our restriction endonuclease analysis on cross I DNA support the sex-specific characteristics of this gene; offspring only expressed the allele of their father (Fig 3B). Further pyrosequencing of the candidate sex-specific imprinted gene revealed that individual brain cDNA expression patterns roughly correspond to patterns indicative of imprinting in females at that SNP locus. In contrast, gene expression in other sampled tissues exhibited an irregular pattern (S2 Table). For the other two reciprocally crossed families (cross III and cross IV), we did not find patterns of genomic imprinting in the gene expression of either male or female offspring brain cDNA (Fig 4C and S2 Table).

### Absence of genomic imprinting in chickens

Genomic imprinting is a phenomenon characterized by parent-of-origin-specific, monoallelic gene expression in offspring. Imprinting exhibits temporal-, tissue- [1], and probably even sex-specificity [35]. However, it is not dependent on parent genotype and does not vary among individuals, because imprinted genes are epigenetically marked and expressed only from the maternal or the paternal allele [2].

Although a previous study confirmed the presence of a parent-of-origin-specific QTL on the chicken genome [17], we were unable to locate any imprinted SNPs in those regions, although we did note some false positives. The lack of evidence for imprinting suggests other reasons for the parent-of-origin characteristics of the QTL, such as paramutation and position-effect variegation [36]. Additionally, we speculate that the generation of false candidate imprinted genes in this study was caused by the random error that occasionally occurs with NGS technology.

Another notable factor that affects our experimental outcome involves the use of a 75% read alignment standard in filtering imprinted SNPs. We chose this strict standard because we have observed that typical imprinted genes in other species are very strongly expressed (e.g., *IGF2* and *H19* gene in mammals) [37–39]. Given that we have identified false positives even with this standard, we felt that a more lenient criterion would yield unreliable results. Although our standard is admittedly arbitrary, we have found that there is currently no set standard in the literature. For example, a previous study on genomic imprinting in the chicken whole embryo chose a fold-change threshold of ≥2.5 [19]. Another study on developing maize endosperm (with two maternal allele copies and one paternal allele) considered gene imprinting to be present if an allele exhibits at least 5× the expression levels of the candidate imprinted allele [12]. Still other research on genomic imprinting employ a statistical test (typically the chi-square) as a threshold [1,16]. Clearly, there is a need for a standard definition of genomic imprinting, in order to facilitate comparison of results across multiple studies.

## Conclusions

Our current results suggest that genomic imprinting is absent in 1-day-old chicken brains. However, our findings are not generalizable across all growth stages and all tissues types, due to the temporal and tissue specificity of genomic imprinting, and the stricter standards we have chosen to impose in our analysis. Further studies in different tissues and at various developmental stages are required to confirm the existence of genomic imprinting in chickens and other oviparous organisms.

## Supporting Information

S1 File Sequencing and alignment quality. This figure shows the overall sequencing quality, we randomly selected one of the re-sequencing libraries to be representative of all the other libraries (Figure A). Quality of the randomly selected RNA-seq library, we found that the base quality was very high, even at the end of reads (Figure B). This figure displays read alignment quality, the horizontal axis shows the proportion of reads aligned to the maternal genome, whereas the vertical axis shows the number of SNPs. Overall, the proportion of aligned reads at most SNP loci was 0.5 (Figure C).
(RAR)

S1 Table Number of reads aligned to parents at each SNP locus. (DOCX)

S2 Table Further pyrosequencing of the most promising candidate imprinted gene in four tissues of individual chickens. (DOCX)
