# Supplementary material for: Next-Generation Sequencing Techniques Reveal that Genomic Imprinting Is Absent in Day-Old Gallus gallus domesticus Brains
Source: PLoS One. 2015 Jul 10;10(7):e0132345. doi: 10.1371/journal.pone.0132345 (PMC4498732; doi:10.1371/journal.pone.0132345)
Supplement: S1 Table — (DOCX) [file pone.0132345.s002.docx]

**S1 Table Number of reads aligned to parents at each SNP locus**

| No.* | In female | | | | | |  | In male | | | | | |
| --- | --- | --- | --- | --- | --- | --- | --- | --- | --- | --- | --- | --- | --- |
|  | CL.C | CL.L | P-value | LC.C | LC.L | P-value |  | CL.C | CL.L | P-value | LC.C | LC.L | P-value |
| 1 | 0 | 49 | 6.37E-11 | 17 | 0 | 3.53E-04 |  | 9 | 33 | 4.11E-03 | 52 | 1 | 1.92E-10 |
| 2 | 26 | 0 | 4.63E-06 | 1 | 18 | 8.47E-04 |  | 30 | 0 | 2.22E-06 | 0 | 28 | 5.16E-06 |
| 3 | 140 | 30 | 1.21E-15 | 8 | 26 | 1.28E-02 |  | 79 | 15 | 3.27E-09 | 15 | 26 | 2.41E-01 |
| 4 | 3 | 19 | 4.95E-03 | 22 | 5 | 7.23E-03 |  | 4 | 17 | 4.66E-02 | 16 | 8 | 3.37E-01 |
| 5 | 11 | 50 | 7.72E-06 | 119 | 0 | 6.58E-26 |  | 17 | 47 | 3.51E-03 | 88 | 60 | 2.35E-13 |
| 6 | 3 | 16 | 1.84E-02 | 43 | 13 | 5.63E-04 |  | 7 | 25 | 1.96E-02 | 29 | 41 | 2.60E-09 |
| 7 | 7 | 34 | 2.54E-04 | 55 | 16 | 4.18E-05 |  | 0 | 23 | 6.05E-05 | 47 | 31 | 3.33E-07 |
| 8 | 34 | 11 | 4.68E-03 | 8 | 37 | 1.57E-04 |  | 25 | 34 | 5.54E-01 | 0 | 39 | 6.85E-09 |
| 9 | 14 | 17 | 8.01E-01 | 13 | 5 | 1.36E-01 |  | 6 | 21 | 4.17E-02 | 18 | 1 | 1.92E-03 |
| 10 | 23 | 5 | 5.01E-03 | 14 | 24 | 2.94E-02 |  | 18 | 1 | 2.09E-03 | 0 | 43 | 3.82E-09 |
| 11 | 0 | 15 | 9.69E-04 | 23 | 0 | 1.93E-05 |  | 23 | 1 | 2.19E-04 | 2 | 16 | 1.27E-02 |
| 12 | 6 | 5 | 8.98E-01 | 11 | 11 | 1.71E-01 |  | 26 | 7 | 1.38E-02 | 8 | 25 | 3.17E-02 |
| 13 | 7 | 11 | 6.15E-01 | 5 | 9 | 6.81E-01 |  | 12 | 1 | 2.78E-02 | 5 | 21 | 2.00E-02 |
| 14 | 15 | 19 | 7.29E-01 | 8 | 45 | 3.16E-04 |  | 12 | 0 | 8.64E-03 | 0 | 40 | 1.63E-08 |
| 15 | 0 | 18 | 2.28E-04 | 11 | 2 | 6.04E-02 |  | \ | \ | \ | \ | \ | \ |
| 16 | \ | \ | \ | \ | \ | \ |  | 19 | 4 | 2.25E-02 | 1 | 14 | 1.09E-02 |

Significant deviation of allelic ratio from 1:1 was evaluated using chi-square tests. P-values were adjusted using a Bonferroni correction.

*The numbers in this column correspond to those in Table 1.

"\" in No. 15 and No. 16 indicate that one of the RNA-seq libraries in the analysis had less than 10 aligned reads.
