# Supplementary material for: Next-Generation Sequencing Techniques Reveal that Genomic Imprinting Is Absent in Day-Old Gallus gallus domesticus Brains
Source: PLoS One. 2015 Jul 10;10(7):e0132345. doi: 10.1371/journal.pone.0132345 (PMC4498732; doi:10.1371/journal.pone.0132345)
Supplement: S2 Table — (DOCX) [file pone.0132345.s003.docx]

**S2 Table Further pyrosequencing on the most promising candidate imprinted gene in four tissues of individual chickens**

| Tissue | Family | Female | | | | Male | | | |
| --- | --- | --- | --- | --- | --- | --- | --- | --- | --- |
|  |  | 1 | 2 | 3 | 4 | 1 | 2 | 3 | 4 |
| Brain | Cross I | 2/98 | 2/98 | 30/70 | 70/30 | 2/98 | 71/29 | 4/96 | 5/95 |
|  | Cross II | 70/30 | 82/18 |  |  | 32/68 | 32/68 | 73/27 | 30/70 |
| Liver | Cross I | 46/54 | 66/34 | 21/79 | 37/63 | 0/100 | 49/51 | 52/48 |  |
|  | Cross II | 68/32 | 63/37 |  |  | 75/25 | 38/62 | 4/96 |  |
| Pectorals | Cross I | 17/83 | 0/100 | 0/100 | 40/60 | 43/57 | 5/95 | 37/63 |  |
|  | Cross II | 69/31 | 37/63 |  |  | 54/46 | 37/63 | 0/100 |  |
| Heart | Cross I | 56/44 | 62/38 | 49/51 | 43/57 | 41/59 | 100/0 | 40/60 |  |
|  | Cross II | Dec-88 | 26/74 |  |  | 38/62 | 56/44 | 47/53 |  |

Values represent the proportion of A/G at the SNP locus. There were 2-4 individuals in each group.
